# Interactional Features of (RO)_4_Ti Species and Their Zr and Hf Analogues

**DOI:** 10.1002/anie.202517522

**Published:** 2025-09-22

**Authors:** Elisabetta Venturin, Rosa M. Gomila, Roberta Bertani, Andrea Pizzi, Paolo Sgarbossa, Arun Dhaka, Antonio Frontera, Giuseppe Resnati

**Affiliations:** ^1^ Department of Chemistry Materials Chemical Engineering “Giulio Natta” Politecnico di Milano via Mancinelli 7 Milano 20131 Italy; ^2^ Department of Chemistry Universitat de les Illes Balears Crta. de Valldemossa Palma de Mallorca (Baleares) 07122 Spain; ^3^ Department of Industrial Engineering University of Padova via F. Marzolo 9 Padova 35131 Italy

**Keywords:** σ‐Hole interactions, Group 4 elements, Hydrogen bond, Titanium catalysts, Zirconium catalysts

## Abstract

Tetroxides of Group 4 elements are benchmark catalysts for aldol reactions and oxidation processes, achieving high yields and regio‐, diastereo‐, and enantioselectivities. Our computational analyses and experimental studies, performed in solution and solid, consistently reveal that these tetroxides form attractive interactions with lone pair compounds exhibiting distinctive σ‐hole bond features. We propose naming these novel bondings as titan bonds (TnBs). These new insights into Ti/Zr/Hf tetroxide interactions may significantly aid in designing more effective catalysts with enhanced selectivities.

## Graphical abstract

Computational and experimental studies reveal that Group 4 tetroxides form a new type of σ‐hole interaction with N and O nucleophiles, which we propose to name “titan bonds” (TnBs). These insights into Ti, Zr, and Hf interactions may aid in the rational design of more selective catalysts for aldol and oxidation reactions.

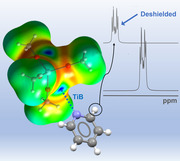

Group 4 derivatives, thanks to the high yields and regio‐, diastereo‐, and enantioselectivities they afford, hold a unique position among Lewis acid catalysts.^[^ ^1^, ^2^ ^]^ Ti(IV) tetroxides^[^ ^3^, ^4^ ^]^ and their Zr/Hf analogues^[^ ^1^, ^5^, ^6^ ^]^ are widely used for carbon–carbon bond formation (aldol condensation) and asymmetric epoxidation of allylic alcohols. Some Hf alkoxides also valorize biomass carbonyls.^[^ ^7^ ^]^

With enantiopure co‐catalysts, Group 4 alkoxides enable highly enantioselective asymmetric aldol reactions^[^ ^1^ ^]^ and kinetic resolution of olefins via epoxidation.^[^ ^2^ ^]^ Oxygen and nitrogen functionalities in co‐catalysts significantly affect selectivities,^[^ ^2^, ^3^, ^4^, ^8^, ^9^ ^]^ consistent with the accepted mechanisms^[^ ^2^, ^4^, ^6^, ^8^, ^9^, ^10^, ^11^ ^]^ which require that substrates and co‐catalysts organize around the metal of the catalyst via Ti/Zr/Hf···N/O interactions.

Characterizing early stages of these interactions in solid and solution is instrumental for rational catalyst design. We report theoretical and experimental evidence that Group 4 alkoxides Z(OR/Ar)_4_ (Z = Ti, Zr, Hf; R = Me, *t*‐Bu, C_6_H_3_R_2_, …) form, with N and O nucleophiles, attractive Z···N/O interactions that are σ‐hole bonds,^[^ ^12^, ^13^, ^14^ ^]^ involving Group 4 elements as the electrophilic atom. In the formed adducts, we also identify weaker N/O–CH···OZ hydrogen bonds (HBs). Following the IUPAC recommendation to designate the elements of a Group from the lightest element, we propose naming these σ‐hole interactions as titan bonds (TnBs).

In this study, ether, tertiary amine, and pyridine derivatives are used as nucleophiles rather than alcohols or primary/secondary amines. This avoids potential substitution of other residue appended to Z by OH or NH moieties and strong N/O–H···OZ HBs (Supporting Information, S.4.1) with tetroxide O atoms that could interfere with the considered Ti/Zr/Hf···N/O short contacts.

We started our studies by calculating the molecular electrostatic potential (MEP) surfaces of Z(OMe)_4_ (Z = Ti, **1a**; Z = Zr, **1b**; Z = Hf, **1c** (Scheme 1). Four symmetric σ‐holes are present at the metals (Figure 1). Notably, the σ‐hole intensities are significantly more positive for Zr and Hf (18.3 and 18.6 kcal·mol^−1^, respectively) than for Ti (7.5 kcal·mol^−1^), indicating a greater potential for directional noncovalent interactions with nucleophiles in the less electronegative Group 4 elements.

**Scheme 1:**
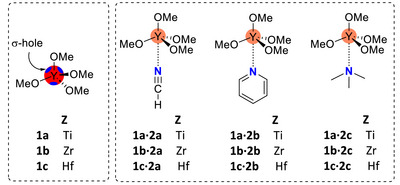
Structural formulas of tetroxides **1a**‐**c** and adducts with lone pair donors **2a‐c**.

**Figure 1:**
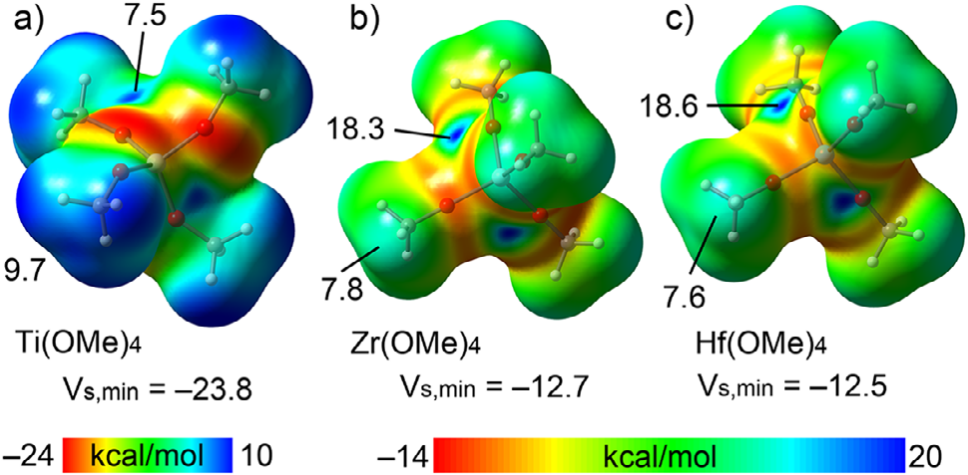
MEP surfaces of **1a** a), **1b** b), and **1c** c). The MEP values at the maximum, minimum, and at the σ‐hole are indicated in kcal·mol^−1^. Isovalue 0.001 a.u.

Tetroxides **1a‐c** were paired with hydrogen cyanide (**2a**, HCN), pyridine (**2b**), and trimethylamine (**2c**), three lone‐pair donors of increasing Lewis basicity. The geometric features of adducts **1a‐c·2a‐c** (Scheme 1) are summarized in Table 1, as well as the calculated binding energies, which range from very weak (−0.5 kcal·mol^−1^ for **1a·2a**) to relatively strong interactions (−18.6 kcal·mol^−1^ for **1c·2c**). As expected, the interaction strengths correlate with both the σ‐hole donor and acceptor strengths: Ti complexes (the weakest σ‐hole donors) and HCN complexes (the weakest acceptors) exhibit the weakest interactions.

**Table 1:** Binding energies (Δ*E*, kcal mol^−1^), equilibrium distances (*d*, Å), O–Z···N angles (α, °), QTAIM parameters (a.u.), and the estimation of the CH···O (*E*_HB_) and Z···N (*E*_Z···N_) energies (kcal mol^−1^) for complexes **1a‐c·2a‐c** (PBE0‐D4/def2‐TZVP level of theory).

	Δ*E*	*E*_HB_	*E*_Z···N_	*d* (Å)	α (°)	ρ	G(r)	V(r)	H(r)	∇^2^ρ
**1a·2a**	−0.5	–	−0.5	2.444	179.8	0.0297	0.0308	−0.0282	0.00267	0.0134
**1b·2a**	−4.8	–	−4.8	2.561	178.5	0.0304	0.0305	−0.0288	0.00169	0.0129
**1c·2a**	−5.2	–	−5.2	2.526	178.7	0.0339	0.0322	−0.0316	0.00061	0.0144
**1a·2b**	−13.5	−4.3	−9.2	2.363	178.8	0.0437	0.0398	−0.0414	−0.00160	0.0153
**1b·2b**	−15.9	−4.0	−11.9	2.477	176.3	0.0444	0.0383	−0.0419	−0.00355	0.0139
**1c·2b**	−16.8	−4.1	−12.7	2.454	176.9	0.0478	0.0392	−0.0444	−0.00518	0.0153
**1a·2c**	−15.2	−5.0	−10.2	2.373	178.9	0.0461	0.0376	−0.0415	−0.00390	0.0135
**1b·2c**	−17.6	−4.0	−13.6	2.503	179.4	0.0449	0.0358	−0.0409	−0.00508	0.0123
**1c·2c**	−18.6	−4.1	−14.5	2.476	179.7	0.0487	0.0370	−0.0440	−0.00700	0.0136

QTAIM parameters (Table 1) are consistent with the trends discussed above. In the HCN complexes **1a‐c·2a**, the total energy densities (H) at the bond critical points (BCPs) are positive and the electron density values (ρ) are below 0.04 a.u., suggesting weak noncovalent interactions. For the remaining complexes **1a‐c·2b,c**, the H values are negative, the |V|/G ratios range from 1.04 to 1.19, the Laplacian values (∇^2^ρ) are positive, and the ρ values slightly exceed 0.04 a.u., these features indicating stronger noncovalent interactions, in agreement with the MEP analyses and the larger binding energies.

Figure 2 displays BCPs and bond paths (BPs) for Ti and Zr adducts (Hf congeners are in Figure S1). In HCN complexes, a single BCP and BP connect the N lone pair [N(sp)] to the metal (Z), confirming the Z···N(sp) interaction. Pyridine and trimethylamine adducts show additional BCPs and BPs for CH···O HBs linking N‐geminal H atoms to methoxy oxygens. Total interaction energies (Δ*E*, Table 1) primarily reflect the Z···N contact in **1a‐c·2a**, while CH···O HBs also contribute in **1a‐c·2b,c**. CH···O HB energies (*E*_HB_), estimated via an empirical correlation,^[^ ^15^ ^]^ range from −4.0 to −5.0 kcal·mol^−1^ (Table 1). The Z···N interaction energies (*E*_Z···N_), ranging from −9.2 kcal·mol^−1^ (**1a·2b**) to −14.5 kcal·mol^−1^ (**1c·2c**), are confirmed as the major driving force, increasing with electron donor basicity and metal σ‐hole depth.

**Figure 2:**
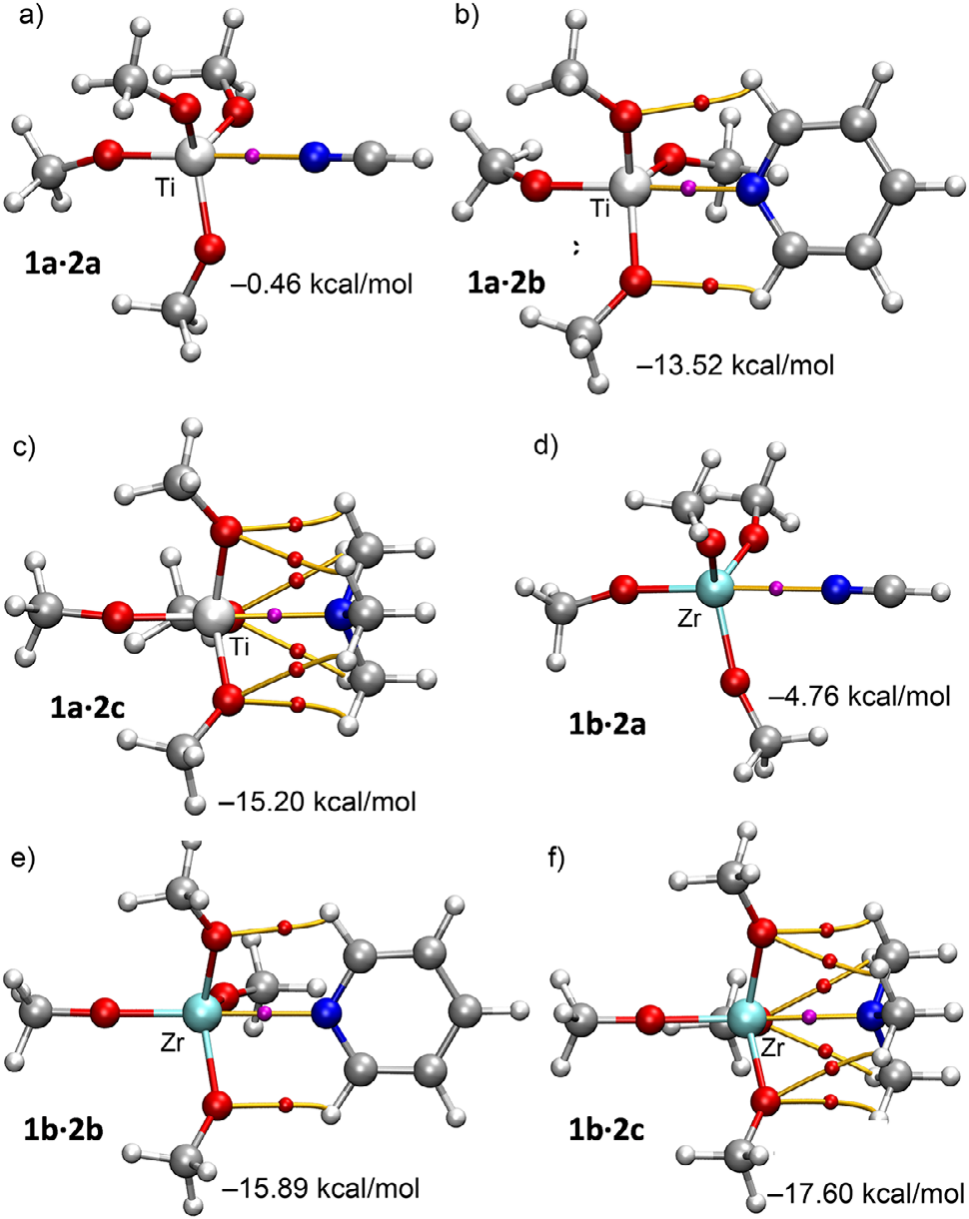
QTAIM distribution of BCPs and BPs in **1a·2a‐c** a)–c) and **1b·2a‐c** d)–f). Only intermolecular BCPs are indicated. The binding energies are indicated.

Further analysis of energetic and QTAIM data (Table 1) reveals a strong correlation between *E*_Z···N_ and the electron density at the corresponding bond critical points (ρ_BCP_) (*r* = 0.943). This allows derivation of an empirical relationship for estimating the strength of the Z···N interaction based on ρ_BCP_: *E*_Z···N_ (kcal·mol^−1^) = −590.8 × ρ_BCP_ (a.u.) + 15.1. This expression should be applied judiciously and only within the range of ρ values used to derive it but may be useful for estimating intramolecular interaction energies (vide infra).

An energy decomposition analysis (EDA) afforded insight into the physical nature of Group 4 σ‐hole interactions. The total interaction energy (*E*_tot_) was partitioned into exchange repulsion (*E*_ex‐rep_), electrostatic (*E*_el_), orbital (*E*_orb_), correlation (*E*_cor_), dispersion (*E*_disp_), and distortion (*E*_dist_)^[^ ^16^ ^]^ components (Table 2).^[^ ^17^ ^]^ Overall, *E*_el_ and *E*_orb_ contributions dominate the interaction. *E*_orb_ is particularly significant in Hf complexes (likely due to the larger and more diffuse atomic orbitals of Hf), while it remains modest relative to *E*_el_ in the case of Ti, consistent with the smaller size of Ti orbitals. Both *E*_cor_ and *E*_disp_ terms increase with the basicity of the nitrogen donor, reflecting stronger noncovalent stabilization.

**Table 2:** EDA (kcal mol^−1^, PBE0‐D4/def2‐TZVP level of theory) for complexes **1a‐c·2a‐c**.

	*E*_tot_	*E*_ex‐rep_	*E*_el_	*E*_orb_	*E*_cor_	*E*_disp_	*E*_dist_
**1a·2a**	−0.5	30.3	−19.7	−10.1	−6.0	−2.1	7.2
**1b·2a**	−4.7	28.0	−18.4	−11.0	−6.0	−2.3	5.0
**1c·2a**	−5.3	31.4	−16.3	−17.0	−6.5	−2.2	5.3
**1a·2b**	−13.5	55.1	−42.5	−18.6	−10.8	−5.3	8.7
**1b·2b**	−15.9	48.2	−37.7	−20.0	−10.2	−5.1	8.9
**1c·2b**	−16.8	51.7	−29.8	−32.9	−10.6	−4.8	9.7
**1a·2c**	−15.3	63.0	−48.2	−20.9	−14.1	−6.1	11.1
**1b·2c**	−17.7	52.9	−40.8	−20.5	−13.1	−5.9	9.8
**1c·2c**	−18.7	56.8	−33.5	−33.8	−13.7	−5.6	11.1

To gain deeper insight into the Z···N interactions from an orbital perspective, we analyzed the complexes exhibiting the highest *E*_orb_ in the EDA via Natural Bond Orbital (NBO) analysis. Figure 3 displays relevant NBOs and second‐order perturbation energies [*E* ^(2)^]. Analysis confirms the expected LP(N) → σ*(Z–O) donor–acceptor interaction, a characteristic feature of σ‐hole bondings.^[^ ^18^ ^]^ Notably large *E* ^(2)^ values (Figure 3) are attributed to significant orbital overlap between the N lone pair and the σ*(Z–O) antibonding orbital.^[^ ^19^ ^]^

**Figure 3:**
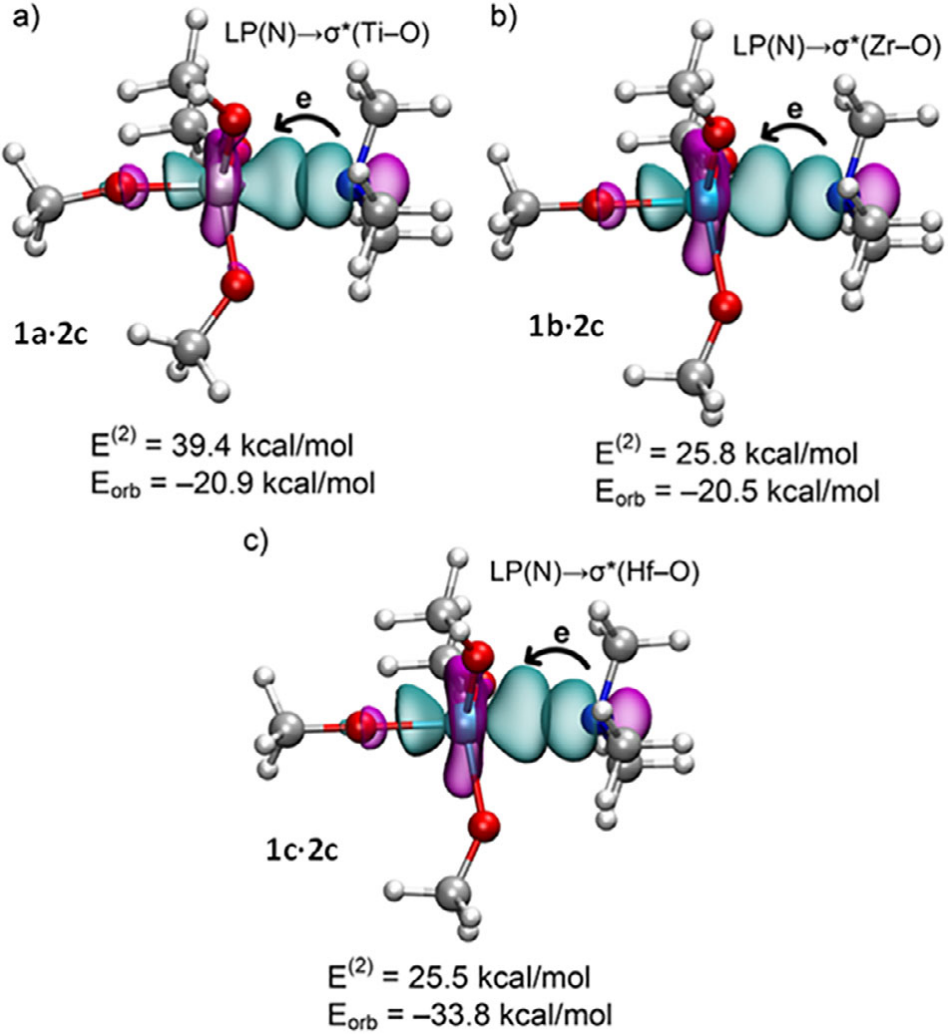
NBOs characterizing the N→σ* electron donation in N(CH_3_)_3_ complexes **1a·2c** a), **1b·2c** b), and **1c·2c** c). The *E* ^(2)^ and *E*_orb_ energies are also indicated.

QTAIM and NBO methods were also performed for two Cambridge Structural Database (CSD) structures of each Group 4 element, wherein Ti/Zr/Hf···N contacts are present. The analyses of the cocrystal between the bis‐(tri‐*t*‐butoxy‐phenoxy)hafnium(IV) compound **1d** (Figure 4) and **2b** (CSD refcode KAPMIZ)^[^ ^20^ ^]^ is described here, while the other Hf structure (refcode LIWZEX)^[^ ^21^ ^]^ and the two structures containing Ti (GOYHAF^[^ ^22^ ^]^ and MAZKOO)^[^ ^23^ ^]^ and Zr (PEMREF^[^ ^24^ ^]^ and TATBAU)^[^ ^25^ ^]^ are discussed in the Supporting Information, S.2.3–S.2.5; Figures S2–S4). In **1d·2b**, two distinct intermolecular Hf···N contacts, with slightly different interaction geometries, are present. The QTAIM and energetic features (Figure 5) are comparable to those of model adduct **1c·2b**. In this structure, the QTAIM analysis also identifies an additional CH···O contact. Based on the ρ values at the BCP, the estimated Hf···N interaction energy is −12.1 kcal·mol^−1^. For computational efficiency, NBO analysis of **1d·2b** was performed using a model comprising half of the adduct. The LP(N) → σ*(Hf–O) donation typical for σ‐hole interaction is identified, and the *E* ^(2)^ value is substantial (41.0 kcal·mol^−1^).

**Figure 4:**
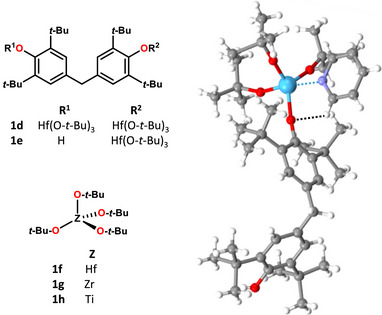
Left: Structural formulas of studied alkoxides **1d**‐**h**. Right: Ball and stick representation of the cocrystal **1e·2b**; TnB and HB are sky‐blue and black dotted lines, color coding: grey, carbon; whitish, hydrogen; red, oxygen; blue, nitrogen; sky‐blue, hafnium.

**Figure 5:**
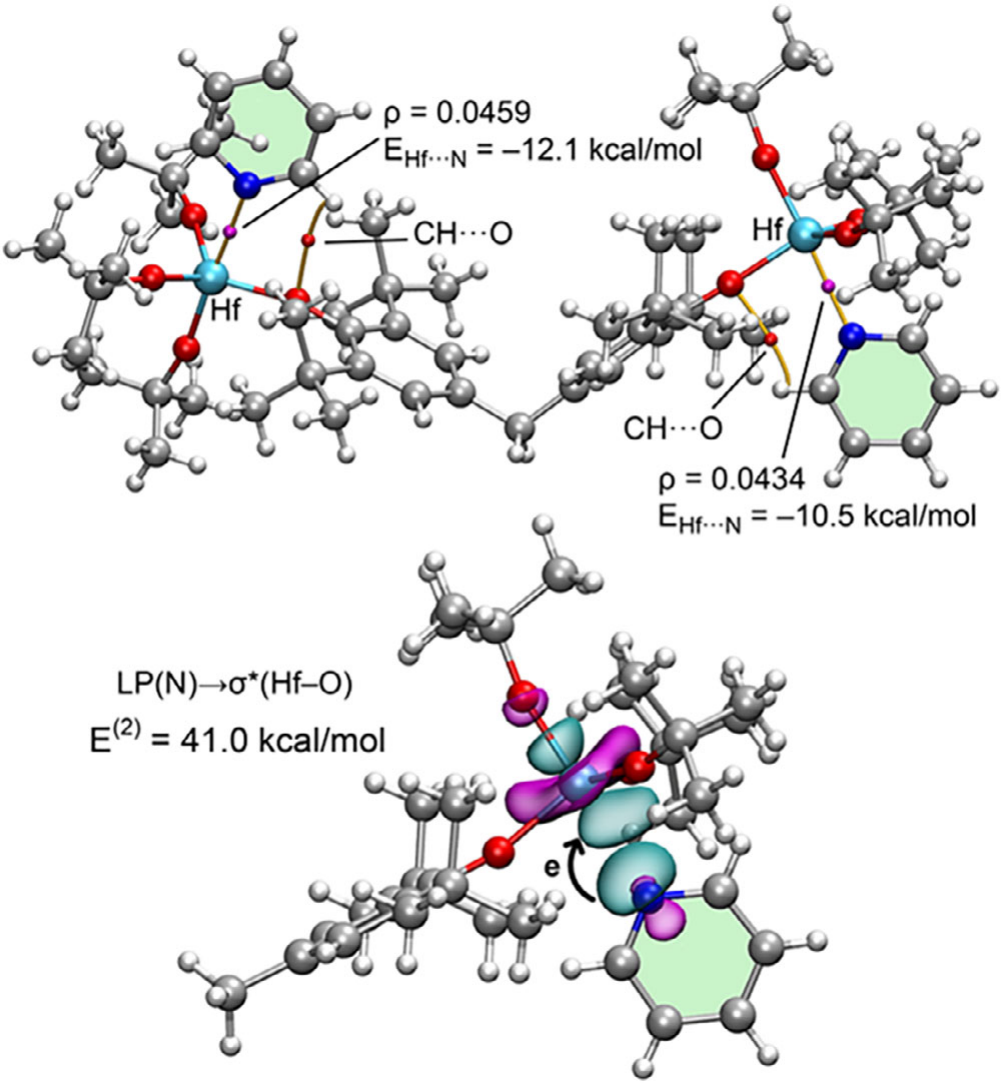
QTAIM analysis (top) and NBOs involved in the LP(N) → σ *(Hf–O) electron donation (bottom) for **1d·2b**.

Experimental studies were started by preparing the mono‐(tri‐*t*‐butoxy‐phenoxy)hafnium(IV) compound **1e** and performing the single crystal X‐ray analysis of its adduct with pyridine **2b** (Figure 4). In the **1e·2b** co‐crystal,^[^ ^26^ ^]^ the N atom forms a short contact with Hf rather than with phenol hydrogen, suggesting a non‐minor acidity of Hf, and the O–Hf···N angle is close to linearity (173.78°). The approach of the nucleophile on the extension of one of the covalent bonds formed by the electrophile is a fingerprint of σ‐hole interactions,^[^ ^18^ ^]^ and the rationalization of the Hf···N bonding as a TnB is supported in the **1e·2b** co‐crystal and in analogous systems in solution. Consistent with calculations on **1a‐c·2b,c** and several CSD structures wherein Ti/Zr,Hf···N/O contacts are present, the pyridine is pinned in its position also via a fairly long (and expectedly weak, as suggested by calculations of model systems **1a‐c·2b**) CH···OHf HB involving one N‐geminal H atom. The presence of prominent and accessible σ‐holes in the experimentally studied compounds **1e‐h** was confirmed by MEP surface analyses (Figure S5), mirroring the features observed in our computational models (Figure 1).

^1^H and ^15^N NMR analyses of solutions of Group 4 alkoxides and N and O nucleophiles were employed to assess the presence of Ti/Zr/Hf···N/O interactions in solution and their σ‐hole nature. In toluene and dichloromethane, two typical solvents of acid‐catalyzed aldol and oxidation reactions,^[^ ^3^, ^5^, ^6^, ^7^, ^8^, ^9^ ^]^ the ^1^H NMR signals of N‐geminal H atoms of pyridine (**2b**), 2‐methyl‐pyridine (**2d**), and quinuclidine (**2e**) are deshielded (Table 3) by the presence of **1d,f,g** (Figure 4). N‐vicinal H atoms behave similarly, but the chemical shift changes (Δδ values) are smaller than for N‐geminal H atoms (Figure 6; Supporting Information S.5.2 and S.5.3). The Δδ values for both proton groups increase with the **1**/**2** ratio. Greater steric hindrance around the metal (**1d** versus **1f**) and the nitrogen (**2d** versus **2b**) causes smaller shift changes, i.e., disfavors association between **1** and **2**. These trends can be explained via the formation, in solution, of the Zr/Hf···N σ‐hole bondings (between metal of **1** and nitrogen of **2**) and the H···O HBs (between protons of **2** and oxygen of **1**) predicted by computation and observed in the solid. The deshielding effect on protons of **2** induced by the donation of electron density from nitrogen to metal prevails over the shielding effect induced by the donation of electron density from oxygen to hydrogen, and protons of **2** move to greater ppm values on **1d,f,g·2** adducts formation.

**Table 3:** Chemical shifts changes of N–CH hydrogen atoms of nucleophiles **2b,d,e** in the presence of Group 4 alkoxides **1d,f‐h** and in C_6_D_5_CD_3_ (black) or CD_2_Cl_2_ (grey) solutions.

	Nucleophile		
Group 4 alkoxide	2b	2d	2e
**1d**	−0.31; −*0.15*	−0.05; −*0.02*	−0.12; −*0.06*
**1f**	−0.59; −*0.26*	−0.30; −*0.10*	−0.44; −*0.23*
	−0.42; −*0.20*	−0.05; ^*a)*^	−0.31;^*b)*^ −*0.04* ^*b)*^
**1g**	−0.56; −*0.26*	−0.23; −*0.07*	−0.44; −*0.24*
	−0.35; −*0.16*	−0.04; ^*a)*^	−0.30;^*b)*^ −*0.05* ^*b)*^
**1h**	0.01; ^*c)*^	^c)^; ^*c)*^	0.01; ^*c)*^

*Note*: In double columns of nucleophiles **2**, the value on the left is Δδ = δ(solution of pure **2**) −δ(solution wherein electrophilic atoms/nucleophilic atoms ratio is 2.0), the value on the right (italic) is Δδ = δ(solution wherein the electrophilic atoms/nucleophilic atoms ratio is 0.50) −δ(solution wherein the electrophilic atoms/nucleophilic atoms ratio is 2.0).

^a)^ Shift to greater ppm value smaller than 0.01.

^b)^ See Supporting Information, S.5.1 for details.

^c)^ Shift to lower ppm value smaller than 0.01.

**Figure 6:**
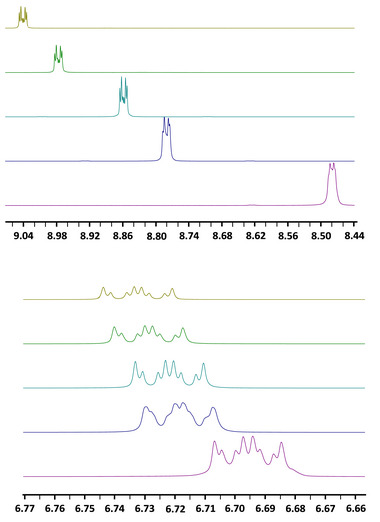
Plotting of ^1^H NMR signals (C_6_D_5_CD_3_ solution) of *ortho* (top) and *meta* (bottom) protons of **2b** in the presence of **1g**. Color coding: **1g/2b** ratio = 2, 1.5, 1.0, and 0.5 yellow, green, teal, and blue, respectively; pure **2b** (same concentration as solution wherein **1g/2b** = 1.0): violet.

The ^1^H NMR signals of **2b,d,e** experience much smaller shifts in the presence of titanium alkoxide **1h** than of zirconium and hafnium analogues **1f,g** and, importantly, they move to smaller ppm values. These differences can be reliably explained by considering the differences between Ti···N and Zr/Hf···N interactions. Consistent with the general tendency of σ‐hole bondings to be weaker when the electrophile electronegativity is greater, calculations predict that Ti···N interactions are remarkably weaker than Zr/Hf···N interactions.^[^ ^27^ ^]^ The transfer of electron density, and the associated deshielding of ^1^H NMR signals, is thus smaller for adducts formed by **1h** than by **1f,g**, even more so as the relevance of *E*_orb_ with respect to other interaction components is modest for Ti and significant for Hf (Table 2). The shielding of ^1^H NMR signals resulting from H···O HBs, thus slightly overcomes the deshielding resulting from Ti···N TnBs and protons of **2** slightly move to lower frequencies on **1h·2b,d,e** adducts formation.

^15^N NMR analyses confirm that in **1·2** adducts the N atoms of **2** act as nucleophiles and donate electron density to the metals of **1**. ^15^N signals move to smaller ppm values when nitrogen interacts with electrophilic transition metals, Lewis acidic Zr and Hf included.^[^ ^28^, ^29^ ^]^ The chemical shift values of the N signal of pyridine **2b** are 11.20, 15.22, and 15.70 ppm smaller when **1d**, **1g**, and **1f** are present (Figure 7; Supporting Information, S.5.4). The corresponding shifts of 2‐methyl‐pyridine (**2d**, conditions as above) are 1.96, 4.44, and 5.05 ppm, confirming the ^1^H NMR indication that association between **1** and **2** decreases when steric hindrance around nitrogen increases.

**Figure 7:**
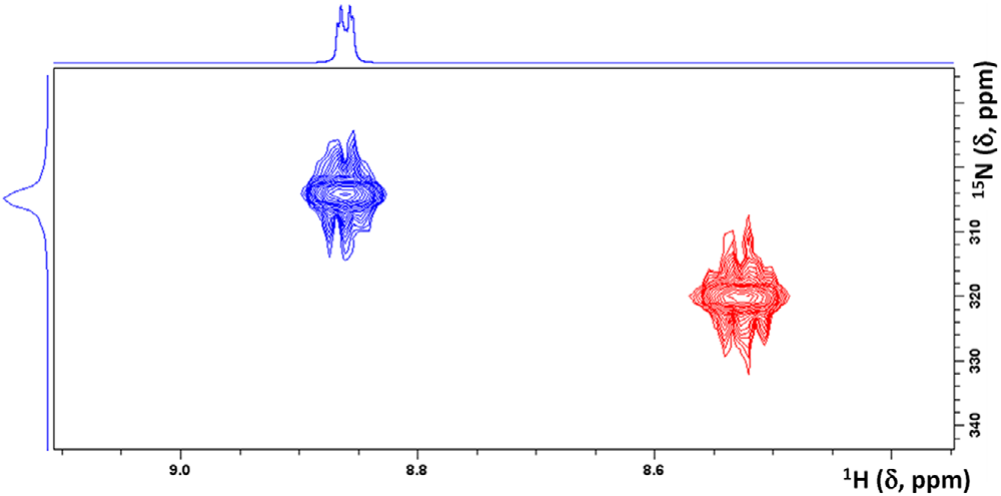
^1^H–^15^N HMBC spectra (N–CH signal, C_6_D_5_CD_3_ solution); blue, **1f·2b** adduct (electrophilic atoms/nucleophilic atoms ratio = 0.5); red, pure **2b**.

To evaluate the general ability of tetraoxides **1** to act as electrophiles, the formation of Ti/Zr/Hf···O interactions in solution was assessed. Zirconium and hafnium alkoxides **1g** and **1f** cause deshielding of ^1^H NMR signals of tetrahydrofuran (**2f**) (Δδ values of OCH_2_ are 0.06 and 0.05 ppm for CD_2_Cl_2_ solutions wherein **1f**/**2f** and **1g**/**2f** ratios are 1). Interestingly, also, titanium alkoxide **1h** causes deshielding, while it is smaller than zirconium and hafnium analogues (Supporting Information, S.5.3). Chemical shift changes increase with the **1**/**2** ratio and are larger for O‐geminal H atoms than for O‐vicinal ones. Clearly, Ti/Zr/Hf···O interactions have features similar to Ti/Zr/Hf···N interactions and can thus be rationalized as TnBs.

Finally, for all examined systems, isochronous protons show a single sharp signal also in the presence of stoichiometric excesses of nucleophile **2**, i.e., at room temperature, the association equilibria are rapid at the NMR timescale, and signals of bonded and unbonded species coalesce. This is a typical and distinctive feature of σ‐hole interactions that differentiates these bondings from coordinative covalent bonds.^[^ ^18^, ^30^ ^]^

In conclusion, calculations and experimental findings consistently prove that the interactions between Ti, Zr, or Hf tetroxides and lone pair‐possessing atoms can be classified as a new family of σ‐hole interactions wherein Group 4 transition metals are the electrophile. Matching the IUPAC term for the Group 4 elements, it is proposed to name TnBs the σ‐hole interactions wherein Group 4 transition metals are the electrophile.

Many transition metals can act as the electrophile in σ‐hole interactions, and these bonds have been presented as “the next frontier in the study of noncovalent bonding.”^[^ ^31^ ^]^ All these σ‐hole bonds share some common aspects and differ from each other in some other aspects. To acknowledge these differences, specific names have been proposed to designate the interactions formed by each Group. For instance, the σ‐hole bondings wherein the electrophile is an element of the Groups 5, 7, 8, and 11 have been named erythronium bond (EyB),^[^ ^32^ ^]^ matere bond (MaB),^[^ ^33^ ^]^ osme bond (OmB),^[^ ^28^ ^]^ and regium bond (RiB),^[^ ^34^ ^]^ respectively.^[^ ^35^ ^]^

An analysis of CSD structures reveals some of the distinctive features of these different σ‐hole bonds. For instance, when the CSD is interrogated on the tetraoxygenated and tetrahedral derivatives of transition metals, it appears that the perrhenate and permanganate derivatives, namely the compounds formed by the heaviest and lightest Group 7 elements, have the highest and lowest propensity to form MaBs, and the pertechnetate systems have an intermediate propensity.^[^ ^33^ ^]^ This trend matches the depth of the transition metals' σ‐holes that become more positive moving from permanganate to perrhenate. Similarly, σ‐holes of tetroxides of Group 8 increase in the order FeO_4_ < RuO_4_ < OsO_4_, and in several CSD structures OsO_4_ forms OmBs with pyridine and amine donors of electron density.^[^ ^28^ ^]^ Tetroxides of Group 4 elements behave similarly, with Ti derivatives showing the less positive σ‐holes and the minor tendency to form σ‐hole interactions. Differently, the same increase of σ‐holes depths is shown by orthovanadate, ‐niobate, and ‐tantalate moieties, but CSD analyses show that orthovanadate derivatives are quite prone to form EyBs, while the derivatives containing the orthoniobate and ‐tantalate moieties, i.e., the analogus compounds formed by the heavier Group 5 elements, seem less prone to give EyBs. This is likely due to the fact that they prefer to interact with nucleophiles via coordinative covalent bonds.^[^ ^32^ ^]^ Similarly, in square planar anions of Group 10 elements, the tetrachloridocuprate species show a much higher propensity than tetrachloridoaurate species to form RiB.^[^ ^34^ ^]^

The tetroxides of Group 4 elements are benchmark catalysts in aldol and oxidation reactions. The new insights reported here on the nature of the early stages of interactions between Group 4 elements and N and O derivatives will help in the rational design of catalysts affording higher selectivities.

## Conflict of Interests

The authors declare no conflict of interest.

## Supporting information



Supporting Information

## Data Availability

The data that support the findings of this study are available in Supporting Information of this article.
