# Supplementary material for: Interactional Features of (RO)4Ti Species and Their Zr and Hf Analogues
Source: Angew Chem Int Ed Engl. 2025 Sep 22;64(45):e202517522. doi: 10.1002/anie.202517522 (PMC12581999; doi:10.1002/anie.202517522)
Supplement: Supplementary file 1 — Supporting Information [file ANIE-64-e202517522-s001.docx]

**SUPPORTING INFORMATION**

Interactional Features of (RO)_4_Ti species

and their Zr and Hf Analogues

Elisabetta Venturin,^[a]^ Rosa M. Gomila,^[b]^ Roberta Bertani,^[c]^ Andrea Pizzi,^[a]^ Paolo Sgarbossa,^[c],^* Arun Dhaka,^[a]^ Antonio Frontera,^[b],*^ and Giuseppe Resnati^[a],*^

[a] E. Venturin, Prof. Dr. A. Pizzi, Dr. A Dhaka, Prof. Dr. G. Resnati
Department of Chemistry, Materials, Chemical Engineering “Giulio Natta”
Politecnico di Milano
via Mancinelli 7, 20131, Milano, Italy
E-mail: G.R. [giuseppe.resnati@polimi.it](mailto:giuseppe.resnati@polimi.it)

[b] Dr. R. M. Gomila, Prof. Dr. A. Frontera
Department of Chemistry
Universitat de les Illes Balears,
Crta. de Valldemossa, 07122 Palma de Mallorca (Baleares), Spain

E-mail: A.F. [toni.frontera@uib.es](mailto:toni.frontera@uib.es)

[c] Prof. Roberta Bertani, Prof. P. Sgarbossa
Department of Industrial Engineering
University of Padova

via F. Marzolo 9, 35131 Padova, Italy
E-Mail: P.S. [paolo.sgarbossa@unipd.it](mailto:paolo.sgarbossa@unipd.it)

**Table Of Content:**

**Page**

**S1. Materials and methods……………………….…………………………………………..… 3**

**S.1.1 Materials……………………………………………………………….….…........ 3**

**S.1.2 Preparation of CH_2_-[3,5-(*t*-Bu)_2_-4-(O-(Hf-(*t*-BuO)_3_)C_6_H_2_]_2_ (1d)...………..… 3**

**S.1.3 Preparation of adduct between tri-*t*-butoxy-(4-(3,5-di-*t*-butyl-4-**

**hydroxybenzyl)-2,6-di-*t*-butylphenoxy)-hafnium(IV) (1e) and pyridine (2b)…………………………………………………………………………….…… 3**

**S2. Computational studies………..………………………………………………………….… 4**

**S.2.1 Generalities………………………………………………………………………. 4**

**S.2.2 QTAIM analysis of 1c·2a-c………………………………………..…………… 4**

**S.2.3 QTAIM and NBO analyses of (tris(2-hydroxy-3,5-di-*t*-butylbenzyl)amine)-**

**isopropoxy-hafnium(IV) (CSD refcode LIWZEX)……………….…............ 5**

**S.2.4 QTAIM and NBO analyses of ((*N*,*N*,*N*-tris(3,5-di-*t*-butyl-2-**

**oxybenzyl)amine)-butoxy-zirconium(IV) (CSD refcode PEMREF) and of**

**(2,4-di-t-butyl-6-({3-[3,5-di-t-butyl-2-oxidophenyl]hexahydroimidazo[1,5-**

**a]pyridin-2(3H)-yl}methyl)phenolato)-bis(2-methylpropan-2-olato)-**

**zirconium(iv) (CSD refcode TATBAU)………………………………………. 6**

**S.2.5 QTAIM and NBO analyses of ((μ-2,2'-(piperazine-1,4-diyl)bis(4,6-di-*t*-**

**butylphenolato))-hexakis(isopropanolato)-di-titanium(IV)) (CSD refcode**

**GOYHAF) and of ((*N*-benzyl-bis(2-methyl-2-oxypropyl)amine-*N*,*O*,*O'*)-**

**bis(isopropoxy)-titanium(IV)) (CSD refcode MAZKOO)..………………… 7**

**S.2.6 MEP surfaces of compounds 1e-1h…………………………………….….… 8**

**S3. Crystallographic details for adduct 1e·2b…………….……..…..……….................... 9**

**S4. Cambridge Structural Database (CSD) searches……………………….……………. 10**

**S.4.1 Selected (Ar/Alk–O)_4_Y (Y = Hf, Zr, Ti) with hydrogen bond(s)…………….. 10**

**S5. NMR Data……………………………………………………………………..……………... 11**

**S.5.1 Details on ^1^H NMR spectra of quinuclidine (2e) in CD_2_Cl_2_………………… 11**

**S.5.2 Selected ^1^H NMR chemical shifts of adducts 1·2 in solution……………... 11**

**S.5.3 Selected ^1^H NMR peaks of adducts 1·2 in solution…………………………. 13**

**S.5.4 Selected ^15^H NMR ………………………………………………………………… 24**

**S6. Cartesian coordinates………………………………………………………….….………. 27**

**S7. References………..…………………………………………………………………………. 33**

**S1. Materials and methods**

**S.1.1 Materials**

All the compounds used in this paper were handled with rigorous exclusion of air and water employing standard Schlenk line and glovebox MBraun MB-10-Compact (nitrogen) techniques. All non-deuterated solvents were stored under nitrogen and used as received (Aldrich). 4,4’-Methylene-bis(2,6-di-*t*-butylphenol), pyridine (**2b**) and quinuclidine (**2e**) were purchased from Sigma-Aldrich. (*t*-BuO)_4_Hf (**1f**) and (*t*-BuO)_4_Zr (**1g**) were purchased from Strem Chemicals, Inc., (*t*-BuO)_4_Ti (**1h**) was purchased from abcr GmbH, 2-methylpyridine (**2d**) was purchased from TCI. All these chemicals were used without further purification. C_6_D_5_CD_3_ and CD_2_Cl_2_ were purchased from Deutero GmbH.

^1^H NMR and HMBC ^1^H-^15^N spectra were recorded at ambient temperature on a Nuclear Magnetic Resonance 600 Bruker Neo spectrometer, equipped with a Prodigy TCI cryo probe, working at 600.13 MHz frequency for ^1^H nucleus. ^1^H chemical shifts were referenced to TMS, while ^15^N chemical shifts were referenced indirectly to TMS, based on the absolute frequency ratios. Data processing and spectra analyses were done using TOPSPIN 3.7 (Bruker BioSpin GmbH, Rheinstetten, Germany) and MestreNova 12.0.2 (Mestrelab Research).

**S.1.2 Preparation of CH_2_-[3,5-(*t*-Bu)_2_-4-(O-(Hf-(*t*-BuO)_3_)C_6_H_2_]_2_ (1d)**

Two equivalents of (*t*-BuO)4Hf (**1f**, 215 μL, d=1.166 mg/μL, 0.531 mmoL) were slowly added to a stirred solution of 4,4’-methylenebis(2,6-di-*t*-butylphenol) (H2-4DBP, 0.113 g, 0.266 mmol) in toluene (2 mL). The colourless reaction mixture immediately turned yellow. After stirring for 12 h, the flask was opened and any volatile was slowly evaporated at room temperature. ^1^H NMR spectrum of the crude reaction product was identical to that reported in ref. [20, main text] of the main manuscript and revealed nearly quantitative yields were obtained.

**S.1.3 Preparation of adduct between tri-*t*-butoxy-(4-(3,5-di-*t*-butyl-4-hydroxybenzyl)-2,6-di-*t*-butylphenoxy)-hafnium(IV) (1e) and pyridine (2b)**

A modified procedure of that described above for **1d** was used wherein one equivalents of (*t*-BuO)_4_Hf (**1f**) per equivalent of H2-4DBP was employed. After stirring for 12 h, the flask was opened and any volatile slowly evaporated at room temperature. Pyridine (**2b**) (2 mL) was added to the residue under stirring and the resulting solution was left to evaporate at room temperature. When first crystals were formed, they were collected and their single crystal X-ray analyses revealed they were the cocrystal **1e·2b**.

**S2. Computational Studies**

**S.2.1 Generalities**

All geometry optimizations of model complexes 1–9 were performed using density functional theory (DFT) at the PBE0-D4/def2-TZVP level of theory, as implemented in the TURBOMOLE 7.7 program package [1-3]. The PBE0 functional combines Perdew–Burke–Ernzerhof (PBE) exchange with exact Hartree–Fock exchange [4], and the D4 dispersion correction was applied to account for long-range interactions [5]. The def2-TZVP basis set was employed for all atoms [6]. For these systems, all complexes were fully optimized in the gas phase without symmetry constraints. The interaction energy was decomposed using the Kitaura-Morokuma energy decomposition analysis (EDA) scheme [7] within the DFT framework as implemented by Ziegler and Rauk [8].

To gain insight into the bonding characteristics, quantum theory of atoms in molecules (QTAIM) analyses were performed using the Multiwfn 3.8 program [9]. The QTAIM graphs and bond critical points (BCPs) were visualized with VMD software [10]. Natural Bond Orbital (NBO) analysis was carried out using the NBO 7.0 program [11]. This allowed for characterization of donor–acceptor interactions, particularly LP(N) → σ*(Y–O) delocalizations relevant to σ-hole bonding. NBO orbital representations were also generated using VMD. Molecular Electrostatic Potential (MEP) surfaces were visualized using GaussView 6.1 [12] using the cubes generated with Multiwfn to identify regions of positive electrostatic potential (σ-holes) around the group 4 metal centers. For the analysis of experimental structures, the selected X-ray geometries were subjected to single-point energy calculations at the same PBE0-D4/def2-TZVP level, without further optimization, to ensure direct comparison with the crystallographic data.

**S.2.2 QTAIM analysis of 1c·2a-c**

The QTAIM analysis of complexes **1c·2a-c** is represented in Figure S1. In HCN complex **1c·2a**, a single BCP and BP connect the N lone pair [N(sp)] to the Hf, confirming the Hf···N(sp) interaction. Pyridine and trimethylamine adducts show additional BCPs and BPs for CH···O HBs linking N-geminal H atoms to methoxy oxygens. IN particular two CH···O contacts for pyridine and 6 BCPs contacts for trimethylamine. The relative contributions of both interactions are summarized in Table 1 (main text).


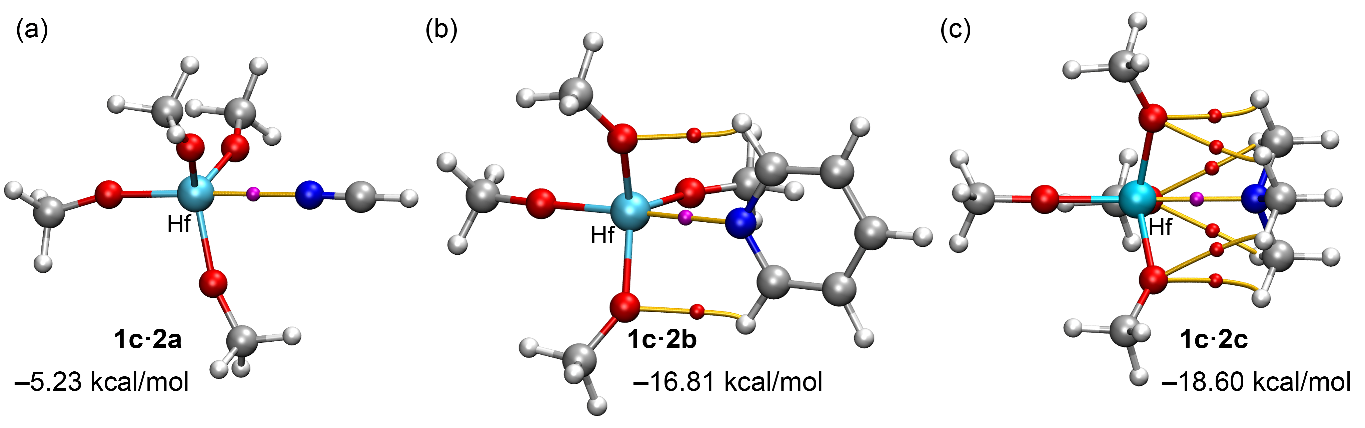


**Figure S1.** QTAIM distribution of BCPs and bond paths in **1c·2a-c** (a-c). Only intermolecular BCPs are indicated. The binding energies are indicated.

**S.2.3 QTAIM and NBO analyses of (tris(2-hydroxy-3,5-di-*t*-butylbenzyl)amine)-isopropoxy-hafnium(IV) (CSD refcode LIWZEX)**

LIWZEX features an intramolecular Hf···N contact, where geometric constraints lead to a significantly shorter distance. This is reflected in a higher ρ value at the BCP (0.0537 a.u.), which exceeds that of complex **1c·2c**, and corresponds to a stronger interaction energy of –16.6 kcal·mol⁻¹.

NBO analysis was performed revealing the expected LP(N) → σ*(Hf–O) donation, characteristic of σ-hole interactions. The second-order perturbation energy is substantial, amounting to 48.4 kcal·mol⁻¹ consistent with the short Hf···N distance in LIWZEX.


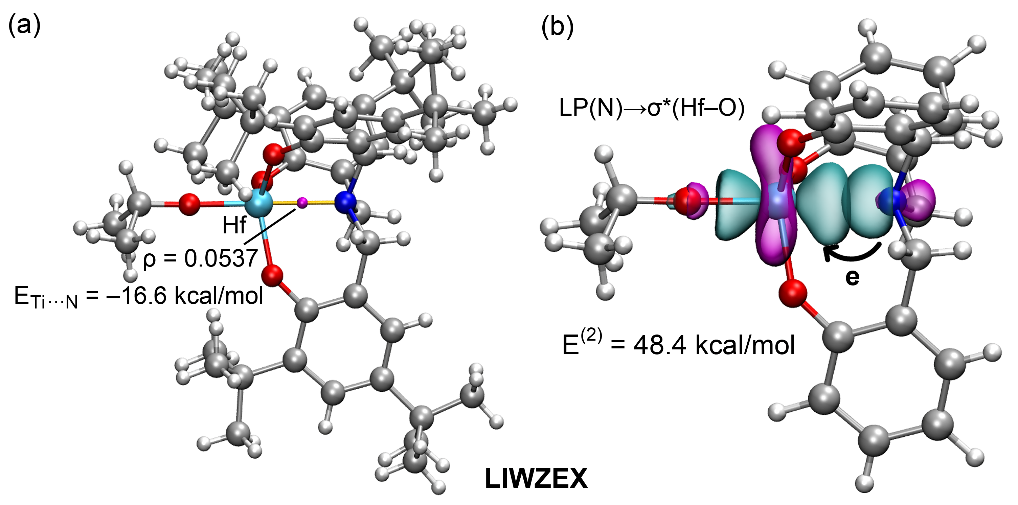


**Figure S2.** (a) QTAIM distribution of BCPs and bond paths in LIWZEX. Only the BCPs and BPs characterizing the titan bond are indicated. The binding energy is indicated. (b) NBOs characterizing the N→σ* electron donation in a reduced model LIWZEX. The E^(2)^ energy is also indicated.

**S.2.4 QTAIM and NBO analyses of ((*N*,*N*,*N*-tris(3,5-di-*t*-butyl-2-oxybenzyl)amine)-butoxy-zirconium(IV) (CSD refcode PEMREF) and of (2,4-di-t-butyl-6-({3-[3,5-di-t-butyl-2-oxidophenyl]hexahydroimidazo[1,5-a]pyridin-2(3H)-yl}methyl)phenolato)-bis(2-methylpropan-2-olato)-zirconium(IV) (CSD refcode TATBAU)**

QTAIM and NBO methods were also applied to two structures exhibiting Zr···N contacts PEMREF and TATBAU, both presenting intramolecular contacts, QTAIM and energetic features (Figure S3) of the Zr···N contacts are comparable to model adduct **1b·2c** (–13.6 kcal/mol, see Table 1, main text). NBO analyses of reduced models of PEMREF, and TATBAU confirmed the expected LP(N) → σ*(Zr–O) donation for σ-hole interaction, with a substantial E(2) value (35.9 and 38.9 kcal·mol⁻¹, respectively), in line with the intramolecular nature of these interactions and short distances.


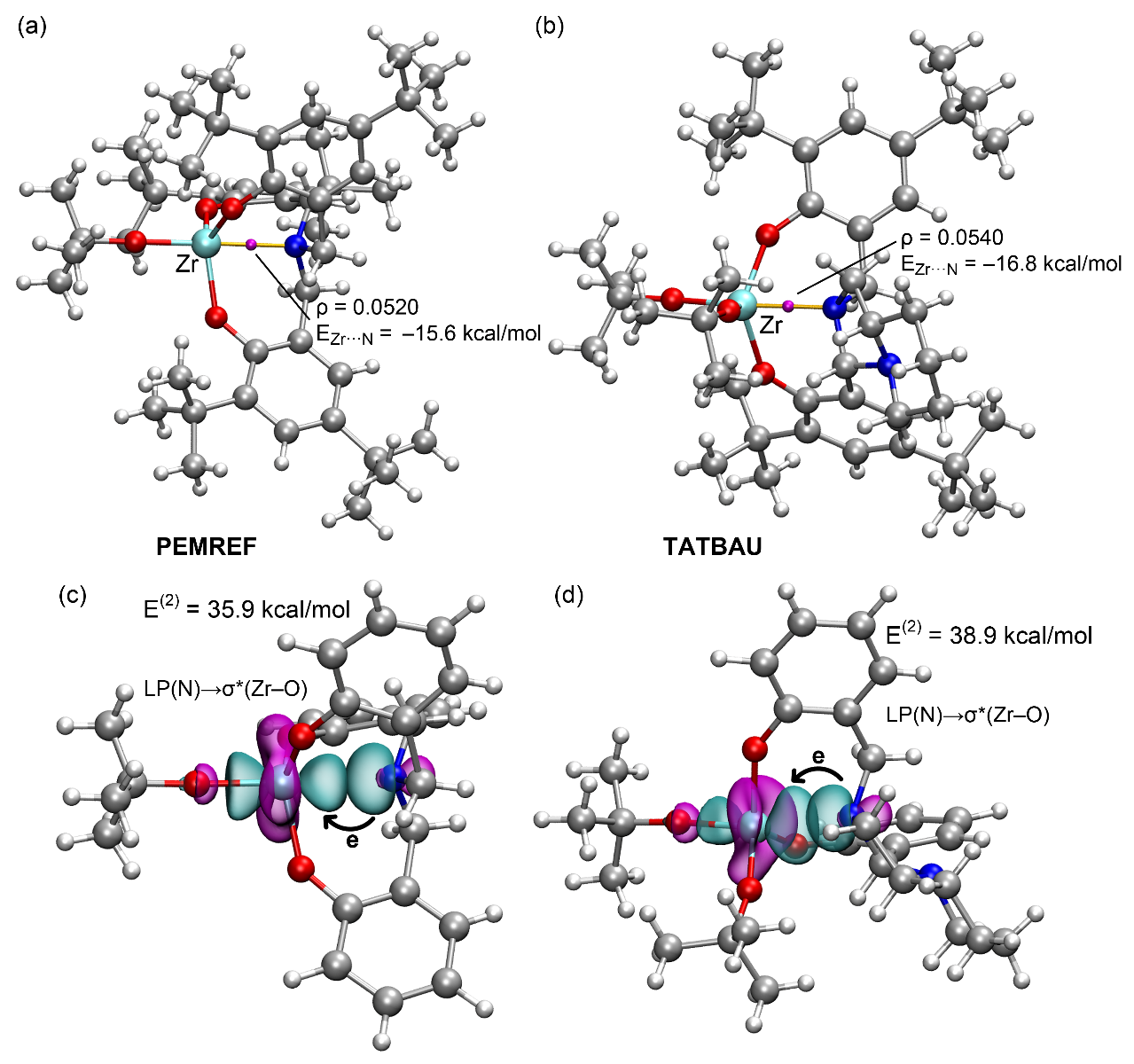


**Figure S3.** QTAIM distribution of BCPs and bond paths in PEMREF (a) and TATBAU (b). Only the BCPs and BPs characterizing the titan bond are indicated. The binding energies are indicated. NBOs characterizing the N→σ* electron donation in reduced models of PEMREF (c) and TATBAU (d). The E^(2)^ energies are also indicated.

**S.2.5 QTAIM and NBO analyses of ((μ-2,2'-(piperazine-1,4-diyl)bis(4,6-di-*t*-butylphenolato))-hexakis(isopropanolato)-di-titanium(IV)) (CSD refcode GOYHAF) and of ((*N*-benzyl-bis(2-methyl-2-oxypropyl)amine-*N*,*O*,*O'*)-bis(isopropoxy)-titanium(IV)) (CSD refcode MAZKOO)**

QTAIM and NBO methods were also applied to two structures exhibiting Ti···N contacts GOYHAF and MAZKOO, both presenting intramolecular contacts, QTAIM and energetic features (Figure S4) of the Ti···N contacts are around 3 kcal·mol⁻¹ weaker than the model adduct **1a·2c** (–10.2 kcal/mol, see Table 1, main text), likely due to geometric restrictions of the intramolecular contacts. NBO analyses of a reduced model of GOYHAF (half of the molecule), and TATBAU (entire model) confirmed the expected LP(N) → σ*(Ti–O) donation for σ-hole interaction, with a substantial E^(2)^ value (35.0 and 37.5 kcal·mol⁻¹, respectively), in line with the those observed for PEMREF and TATBAU (see Figure S3).


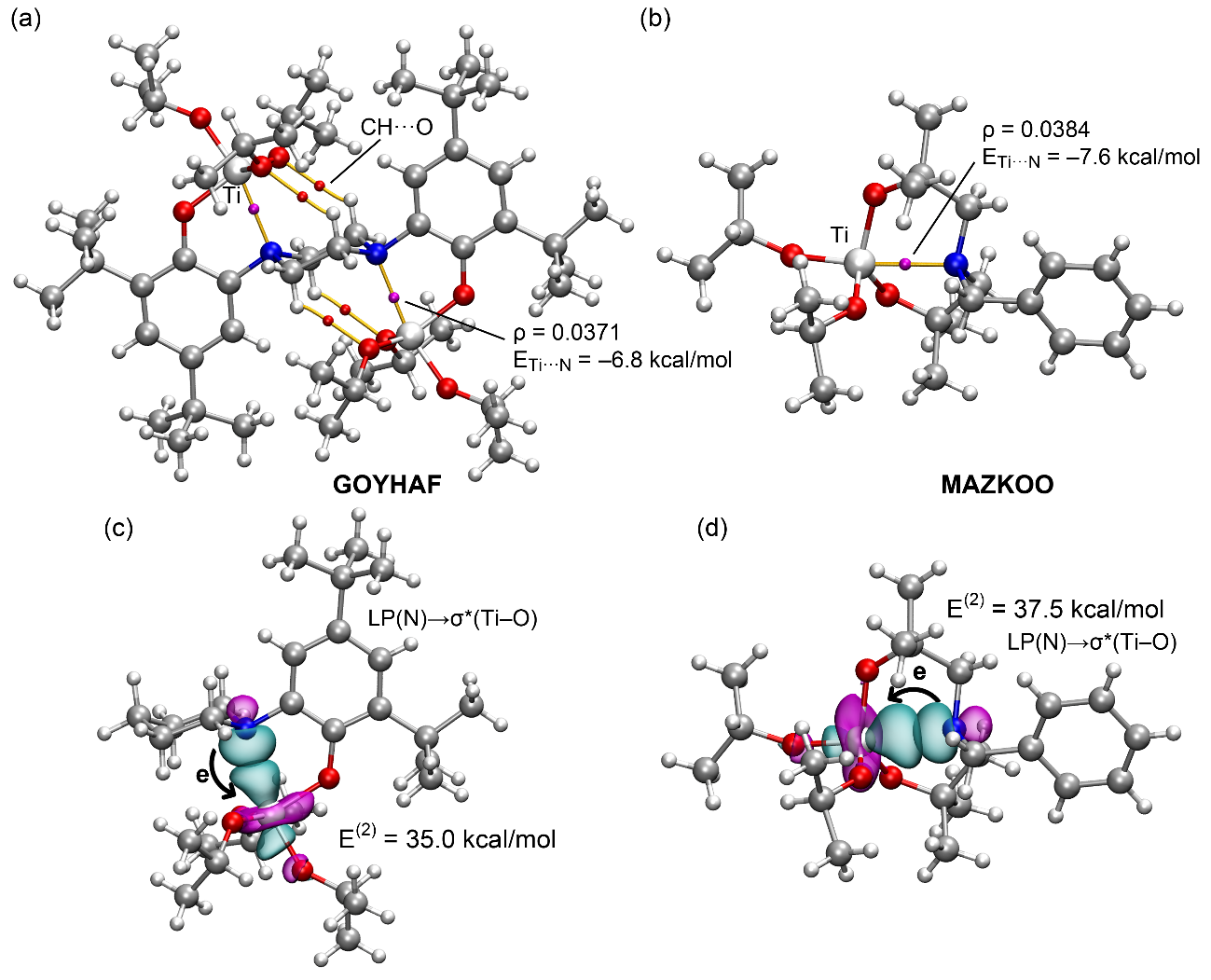


**Figure S4.** QTAIM distribution of BCPs and bond paths in GOYHAF (a) and MAZKOO (b). Only the BCPs and BPs characterizing the titan bond are indicated. The binding energies are indicated. NBOs characterizing the N→σ* electron donation in a reduced model of GOYHAF (c) and entire model of MAZKOO (d). The E^(2)^ energies are also indicated.

**S.2.6 MEP surfaces of compounds 1e-1h**

The MEP surfaces for the experimentally studied compounds **1e-1h** are shown in Figure S4. The largest σ-hole, with an intensity of 29.5 kcal/mol, is found on the Hf atom of compound **1e**, likely due to the planar aromatic ring making the σ-hole more accessible. The σ-hole intensities for the Z(*^t^*BuO)₄ compounds are less positive and are comparable to those of the Z(MeO)₄ models used in the theoretical study. These results confirm that the presence of bulkier ligands does not eliminate the electrophilic σ-hole on the Group 4 metal centers, validating our computational approach.


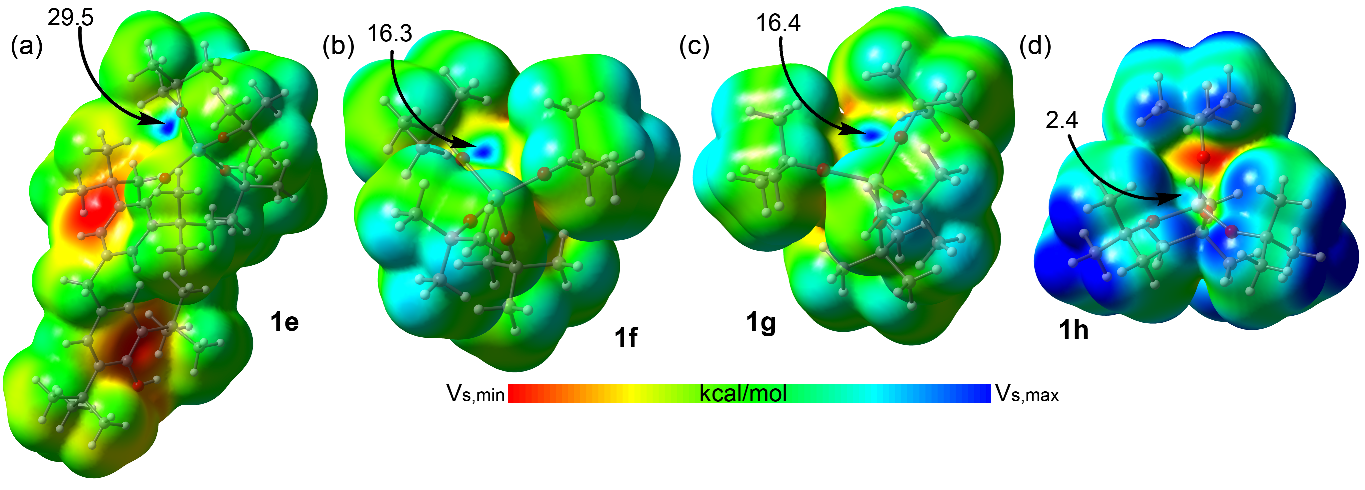


**Figure S5**. MEP surfaces (isovalue 0.001 a.u.) of **1e** (a), **1f** (b), **1g** (c) and **1h** (d). The values at σ-holes are given in kcal/mol

**S3. Crystallographic details for adduct 1e·2b**

The single crystal data were collected using a XtaLAB Synergy diffractometer, equipped with a HyPix detector. Unit cell refinement and data reduction were performed using CrysAlisPro 1.171.41.98a. Structures were solved by direct methods using SHELXT and refined by full-matrix least-squares on F^2^ with anisotropic displacement parameters for the non-H atoms using Olex2. Absorption correction was performed based on multi-scan procedure [13-15].

**Table S1.** Crystal data and structure refinement for adduct **1e·2b**.

| Identification code | **1e·2b** |
| --- | --- |
| Empirical formula | C_46_H_75_HfNO_5_ |
| Formula weight | 900.56 |
| Temperature/K | 100.01(11) |
| Crystal system | triclinic |
| Space group | P-1 |
| a/Å | 9.32030(10) |
| b/Å | 10.28670(10) |
| c/Å | 27.3927(3) |
| α/° | 94.3620(10) |
| β/° | 97.0720(10) |
| γ/° | 115.0810(10) |
| Volume/Å^3^ | 2336.40(5) |
| Z | 2 |
| ρ_calc_g/cm^3^ | 1.280 |
| μ/mm^‑1^ | 4.441 |
| F(000) | 940.0 |
| Crystal size/mm^3^ | 0.134 × 0.114 × 0.008 |
| Radiation | Cu Kα (λ = 1.54184) |
| 2Θ range for data collection/° | 10.58 to 153.384 |
| Index ranges | -11 ≤ h ≤ 11, -12 ≤ k ≤ 12, -33 ≤ l ≤ 34 |
| Reflections collected | 84699 |
| Independent reflections | 9212 [R_int_ = 0.0906, R_sigma_ = 0.0398] |
| Data/restraints/parameters | 9212/0/583 |
| Goodness-of-fit on F^2^ | 1.161 |
| Final R indexes [I>=2σ (I)] | R_1_ = 0.0634, wR_2_ = 0.1739 |
| Final R indexes [all data] | R_1_ = 0.0665, wR_2_ = 0.1755 |
| Largest diff. peak/hole / e Å^-3^ | 4.17/-2.91 |
| Deposition Number | 2469700 |

**S4. Cambridge Structural Database (CSD) searches**

**S.4.1 Selected (Ar/Alk–O)_4_Y (Y = Hf, Zr, Ti) with hydrogen bond(s)**

Selected structures (CSD 2024.1.0 version) wherein one, or more, oxygen atom(s) in (C–O)_4_Y (Y = Hf, Zr, Ti) forms one, or more, hydrogen bond(s) (HBs) with an O–H or N–H residue laying close to Y:

Y = Hf

CATFIP, CATFOV, CATFUB, HUNGAZ, NAYDAS01, OCIXUW, ZIZWAG, ABUPOF.

Y = Zr

CIGCAY, EJOTIH, EJOTON, EJOTUT, EPIJIY, EPIJUK, FAPQOD, JOFJOF, KAWLIE, KURCUY, MAQTEE, MIZWEY, MUHFEB, OCIYAD, QILKED, RENWIR, RENWOX, REWPAN, SIKZOC, SILBAR, SILCAS, TAYVEY, TIYGAK, TUTLOK, OCOKOO, YODBEZ.

Y = Ti

ABORUH, AXEPOK, BABBOY, BOYPUC, CIFQEQ, CIGCEC, CIJROG, CIPFEN, CIRFIR, CIPFOX, CIPFUD, DIRQIH, DOQSIL, EGUQOP. FECHAX, FECHEB, FEQPEX, FEQPIB, FEQPOH, GUBMOG, GUBMUM, GUBNAT, GUBNEX, HUQLOW, JOYBIK, KEMCAI, MAQTII, MAQTUU, MUHDUP, MUHFAX, NIRWER, OCISOL, SONCUW, SONDAD, SORTOL, TATBIC, TATREO, TIYCOU, TIYFAJ, TIYFEN, TIYFIR, TIYFUD, TUTLIE, UKELAX. VECJUK, VESKEN, VIWMIB, VIWMIB, VIWMIB, VIWMIB, WUWWUJ, WUYVEU, WUYVEU01, XEDLUN, XEDMAU, XUJHIU, XUJHOA, XUJHUG, XUJJAO, XUWZOF, XUWZUL.

**S5. NMR data**

**S.5.1 Details on ^1^H NMR spectra of quinuclidine (2e) in CD_2_Cl_2_**

At room temperature, quinuclidine slowly reacts with CD_2_Cl_2_ and affords *N*-chloromethyl-quinuclidinium chloride. The formation of this Menshutkin reaction product was observed also in CD_2_Cl_2_ solutions of **1f-h** and **2e**. As a consequence, Δδ values reported in Table 3 for solutions containing **1f-h·2e** adducts refer to actual concentrations of **2e** lower than the nominal ones (as revealed also by ^1^H NMR signals integration). While a quantitative comparison of these Δδ values with those afforded by **2b,d** cannot thus be made, the qualitative reliability of these Δδ values is confirmed by the fact that the observed low fields shifts increase when the **2e** concentration decreases and are greater for the N–CH_2_ signals than for the N–CH_2_C*H*_2_ ones.

**S.5.2. Selected ^1^H NMR chemical shifts of adducts 1·2 in solution**

Chemical shifts of nucleophiles **2** are reported at different **1/2** ratios in order to show the concentration dependent chemical shifts changes.

The stock solutions of the nucleophiles **2b,d** were prepared by dissolving 1 mL of base with the deuterated solvent to 3 mL in a volumetric flask. Aliquots were added using micropipettes (10-100 uL range). The stock solution of the nucleophile **2e** was prepared by dissolving the base (93 mg, 0.836 mmol) in a volumetric flask to 3 mL with the solvent. Aliquots were added using micropipettes (100-1000 uL range). Prior to usage, CD_2_Cl_2_ was filtered over a neutral Al_2_O_3_ plug (10 mL over 1 g Al_2_O_3_) to remove trace amounts of HCl/DCl, potentially generated by photodegradation of CD_2_Cl_2_.

**CH_2_-[3,5-(*t*-Bu)_2_-4-(O-(Hf-(*t*-BuO)_3_)C_6_H_2_]_2_** **(1d) and pyridine (2b) solutions**

**1d·2b:** (600 MHz, C_6_D_5_CD_3_) δ(ppm): N–CHC*H*:

**1d/2b** ratio = 1: δ = 6.49

**1d/2b** ratio = 0.5: δ = 6.51

**1d/2b** ratio = 0.25: δ = 6.62

Pure **2b** (same concentration as solution wherein **1d/2b** ratio is 1): δ = 6.70

**CH_2_-[3,5-(*t*-Bu)_2_-4-(O-(Hf-(*t*-BuO)_3_)C_6_H_2_]_2_** **(1d) and quinuclidine (2e) solutions**

**1d·2e:** (600 MHz, C_6_D_5_CD_3_) δ(ppm), N–CH_2_C*H_2_*:

**1d/2e** ratio = 1: δ = 1.314 (partial overlap with *t*-BuO signal of **1d**)

**1d/2e** ratio = 0.25: δ = 1.304 (partial overlap with *t*-BuO signal of **1d**)

Pure **2e** (same concentration as solution wherein **1d/2e** ratio is 1): δ = 1.298

**Hafnium(IV) *t*-butoxide (1f) and pyridine (2b) solutions**

**1f·2b:** (600 MHz, C_6_D_5_CD_3_) δ(ppm): N–CHC*H*:

**1f/2b** ratio = 2: δ = 6.73

**1f/2b** ratio = 1: δ = 6.72

**1f/2b** ratio = 0.5: δ = 6.71

Pure **2b** (same concentration as solution wherein **1f/2b** ratio is 1): δ = 6.70

**Zirconium(IV) *t*-butoxide (1g) and quinuclidine (2e) solutions**

(600 MHz, CD_2_Cl_2_) δ(ppm): N–CH_2_C*H_2_*:

**1g/2e** ratio = 2: δ = 1.55

**1g/2e** ratio = 1: δ = 1.54

**1g/2e** ratio = 0.5: δ = 1.53

Pure **2e** (same concentration as solution wherein **1g/2e** ratio is 1): δ = 1.49

**Hafnium(IV) *t*-butoxide (1f) and tetrahydrofurane (2f) solutions**

(600 MHz, CD_2_Cl_2_) δ(ppm): O–CH_2_:

**1/2** ratio = 1: δ = 3.74

**1/2** ratio = 0.5: δ = 3.73

Pure **2f** (same concentration as solution wherein **1f/2f** ratio is 1): δ = 3.68

**S.5.3 Selected ^1^H NMR peaks of adducts 1·2 in solution**

Partial plottings of spectra of nucleophiles **2** are reported at different **1/2** ratios to show the concentration dependent chemical shifts changes.

At all **1/2** ratios, even in the presence of excess **2**, signals are as sharp as those of pure **2** proving the coalescence of signals of bonded and unbonded nucleophiles, namely that the association equilibrium is rapid at the NMR timescale.

**Hafnium(IV) *t*-butoxide (1f) and pyridine (2b) solutions** (600 MHz, C_6_D_5_CD_3_) δ(ppm): NCHC*H*:

**1f/2b** ratio = 2: green

**1f/2b** ratio = 0.5: blue

Pure **2b** (same concentration as solution wherein **1f·2b** ratio is 0.5): violet.

**Zirconium(IV) *t*-butoxide (1g) and 2-methylpyridine (2d) solutions** (600 MHz, C_6_D_5_CD_3_) δ, NCHC*H*:

**1g/2d** ratio = 1: green

Pure **2d** (same concentration as solution wherein **1g/2d** ratio is 1): violet.

**Titanium(IV) *t*-butoxide (1h) and pyridine (2b) solutions** (600 MHz, C_6_D_5_CD_3_) δ(ppm): NCH:

**1h/2b** ratio = 2: yellow

**1h/2b** ratio = 0.5: blue

Pure **2b** (same concentration as solution wherein **1h·2b** ratio is 0.5): violet.

**Hafnium(IV) *t*-butoxide (1f) and quinuclidine (2e) solutions** (600 MHz, C_6_D_5_CD_3_) δ(ppm): NCH_2_C*H_2_*:

**1f/2e** ratio = 2: yellow (partial overlap with *t*-BuO signal of **1f**)

**1f/2e** ratio = 1: green (partial overlap with *t*-BuO signal of **1f**)

**1f/2e** ratio = 0.5: blue

Pure **1f** (same concentration as solution wherein **1f/2e** ratio is 1): brownish

Pure **2e** (same concentration as solution wherein **1f/2e** ratio is 1): violet

**Hafnium(IV) *t*-butoxide (1f) and quinuclidine (2e) solutions** (600 MHz, CD_2_Cl_2_) δ(ppm): NCH_2_C*H_2_*:

**1f/2e** ratio = 2: yellow

**1f/2e** ratio = 1: green

**1f/2e** ratio = 0.5: blue

Pure **2e** (same concentration as solution wherein **1f/2e** ratio is 1): violet

**Zirconium(IV) *t*-butoxide (1g) and pyridine (2b) solutions** (600 MHz, CD_2_Cl_2_) δ(ppm): NCHC*H*:

**1g/2b** ratio = 2: yellow

**1g/2b** ratio = 1: green

**1g/2b** ratio = 0.5: blue

Pure **2b** (same concentration as solution wherein **1g/2b** ratio is 1): violet

**Hafnium(IV) *t*-butoxide (1f) and pyridine (2b) solutions** (600 MHz, CD_2_Cl_2_) δ(ppm): NCHC*H*:

**1f/2b** ratio = 2: yellow

**1f/2b** ratio = 1: green

**1f/2b** ratio = 0.5: blue

Pure **2b** (same concentration as solution wherein **1f/2b** ratio is 1): violet

**Zirconium(IV) *t*-butoxide (1g) and 2-methylpyridine (2d) solutions** (600 MHz, CD_2_Cl_2_) δ(ppm): NCHC*H*:

**1g/2d** ratio = 2: yellow

**1g/2d** ratio = 1: green

**1g/2d** ratio = 0.5: blue

Pure **2d** (same concentration as solution wherein **1g/2d** ratio is 1): violet

**Hafnium(IV) *t*-butoxide (1f) and 2-methylpyridine (2d) solutions** (600 MHz, CD_2_Cl_2_) δ(ppm): NC(CH_3_)C*H* (left), NCHC*H* (right):

**1f/2d** ratio = 2: yellow

**1f/2d** ratio = 1: green

**1f/2d** ratio = 0.5: blue

Pure **2d** (same concentration as solution wherein **1f/2d** ratio is 1): violet

**Titanium(IV) *t*-butoxide (1h) and tetrahydrofurane (2f) solutions** (600 MHz, CD_2_Cl_2_) δ(ppm): OCH_2_:

**1h/2f** ratio = 1: green

**1h/2f** ratio = 0.5: blue

Pure **2f** (same concentration as solution wherein **1h/2f** ratio is 1): violet

**Titanium(IV) *t*-butoxide (1h) and tetrahydrofurane (2f) solutions** (600 MHz, CD_2_Cl_2_) δ(ppm): OCH_2_C*H_2_*:

**1h/2f** ratio = 1: green

**1h/2f** ratio = 0.5: blue

Pure **2f** (same concentration as solution wherein **1h/2f** ratio is 1): violet

**S.5.4 ^15^N NMR**

**^1^H−^15^N HMBC spectra (N–CH signal, C_6_D_5_CD_3_ solution); blue, 1d·2b adduct (electrophilic atoms/nucleophilic atoms ratio = 0.5), red, pure 2b.**


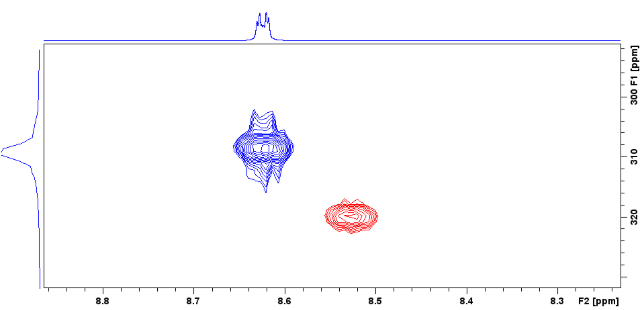


**^1^H−^15^N HMBC spectra (N–CH signal, C_6_D_5_CD_3_ solution); blue, 1g·2b adduct (electrophilic atoms/nucleophilic atoms ratio = 0.5), red, pure 2b.**


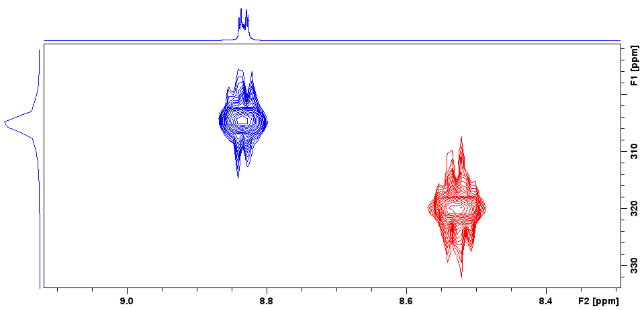


**^1^H−^15^N HMBC spectra (N–CH signal, C_6_D_5_CD_3_ solution); blue, 1d·2d adduct (electrophilic atoms/nucleophilic atoms ratio = 0.5), red, pure 2d.**


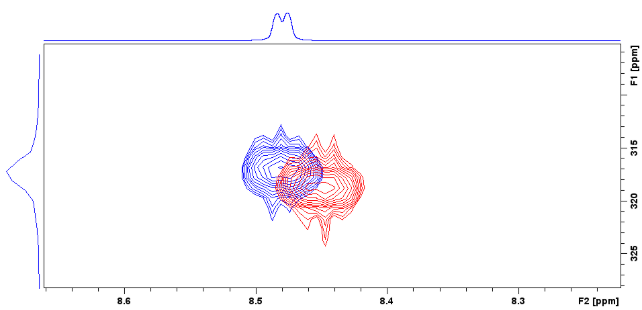


**^1^H−^15^N HMBC spectra (N–CH signal, C_6_D_5_CD_3_ solution); blue, 1f·2d adduct (electrophilic atoms/nucleophilic atoms ratio = 0.5), red, pure 2d.**


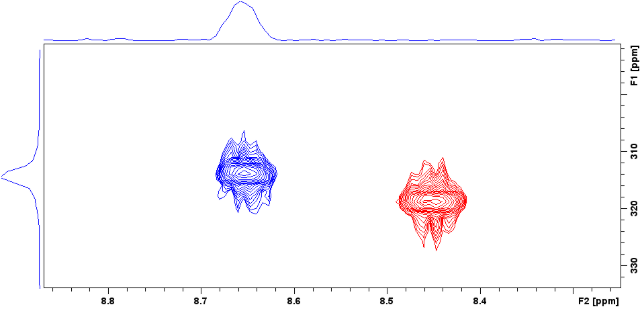


**^1^H−^15^N HMBC spectra (N–CH signal, C_6_D_5_CD_3_ solution); blue, 1g·2d adduct (electrophilic atoms/nucleophilic atoms ratio = 0.5), red, pure 2d.**


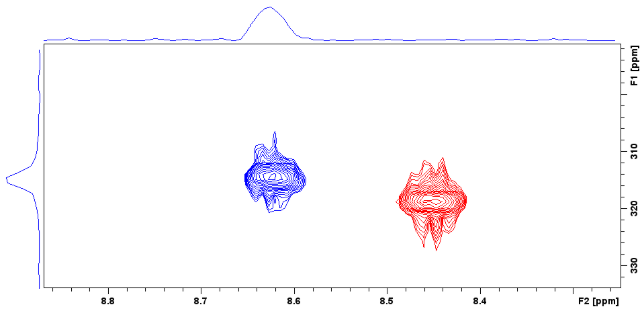


**S6. Cartesian coordinates**

**1a·2a**

Ti 9.3982784 5.1827493 10.5291949

O 8.7486385 5.7982685 8.9443433

O 8.2818110 4.1784411 11.5589768

O 9.4065198 6.6925508 11.4681594

O 11.1725872 4.7807457 10.5649283

C 8.5249662 7.0141449 8.3162219

H 7.6917856 6.9261863 7.6087584

H 8.2729457 7.8044144 9.0351811

H 9.4105652 7.3376087 7.7542890

C 9.5624888 8.0267977 11.8027348

H 8.5939594 8.5396831 11.8069380

H 10.0014146 8.1210958 12.8023080

H 10.2192196 8.5396131 11.0903644

C 12.3080656 5.1819963 11.2529488

H 12.6423275 4.3970666 11.9427898

H 13.1265064 5.3854768 10.5515586

H 12.1277876 6.0926773 11.8396197

C 7.5138664 4.3906285 12.6967092

H 7.7329740 3.6268921 13.4527664

H 7.7112857 5.3761812 13.1385758

H 6.4447981 4.3319919 12.4571432

N 9.3813902 3.1058927 9.2413834

C 9.3746832 2.1387113 8.6370358

H 9.3680000 1.2325937 8.0715972

**1b·2a**

Zr 9.4906932 5.6657281 10.7072141

O 8.9141873 6.0844959 8.8935809

O 8.1613550 4.8572233 11.8840253

O 9.6375641 7.4306769 11.4714483

O 11.3185337 5.0143143 10.8745804

C 8.5999399 6.8392545 7.7791956

H 7.5918784 6.6003547 7.4166755

H 8.6313768 7.9144078 7.9995246

H 9.3044130 6.6438189 6.9605077

C 9.4987836 8.5590716 12.2550897

H 8.8419489 8.3667478 13.1129597

H 10.4709064 8.8902036 12.6406973

H 9.0672928 9.3865008 11.6783318

C 12.6822488 4.9702031 11.0915385

H 12.9436521 4.1543073 11.7775668

H 13.2252977 4.8044806 10.1520914

H 13.0491356 5.9076197 11.5295904

C 7.2475857 4.0043979 12.4666335

H 7.2100114 3.0370806 11.9446326

H 7.5050532 3.8062915 13.5149611

H 6.2373105 4.4328062 12.4451667

N 9.2441468 3.3105629 9.7316731

C 9.1252009 2.2547495 9.3154432

H 9.0183490 1.2671102 8.9213981

**1c·2a**

Hf 9.4901721 5.6573566 10.7031388

O 8.9145643 6.0745548 8.8875465

O 8.1592354 4.8571362 11.8854077

O 9.6382809 7.4239030 11.4670822

O 11.3189739 5.0048967 10.8691826

C 8.5998162 6.8364336 7.7791666

H 7.5915063 6.6008074 7.4149061

H 8.6314617 7.9103727 8.0050256

H 9.3029153 6.6465419 6.9578662

C 9.4987237 8.5506437 12.2514896

H 8.8430730 8.3590526 13.1106943

H 10.4706035 8.8834945 12.6367967

H 9.0659382 9.3788492 11.6764305

C 12.6813460 4.9684022 11.0903402

H 12.9461662 4.1539434 11.7768881

H 13.2291497 4.8055148 10.1530521

H 13.0431410 5.9071549 11.5296150

C 7.2461119 4.0046891 12.4671152

H 7.2070489 3.0366284 11.9462739

H 7.5022983 3.8058426 13.5155567

H 6.2354994 4.4321796 12.4463026

N 9.2502272 3.3350271 9.7378500

C 9.1296698 2.2787099 9.3241167

H 9.0209419 1.2902729 8.9326825

**1a·2b**

Ti 9.4729877 5.1222646 10.5542667

O 9.0055388 5.9819765 9.0275139

O 8.2020538 4.1977232 11.4932407

O 9.5835640 6.5632673 11.6426922

O 11.2182560 4.5757125 10.6512533

C 8.2498481 5.9953207 7.8683489

H 8.7156009 6.6470800 7.1198569

H 8.1628748 4.9909302 7.4326459

H 7.2361461 6.3686517 8.0595132

C 9.5114240 7.9458233 11.5311390

H 9.2403131 8.2426434 10.5100721

H 8.7603849 8.3468632 12.2218796

H 10.4765518 8.4031692 11.7799174

C 12.3150666 4.9806500 11.4053600

H 12.7141872 4.1376187 11.9826347

H 13.1154452 5.3503996 10.7519695

H 12.0479014 5.7807868 12.1067028

C 7.6464127 4.3602196 12.7602622

H 7.9506230 3.5397506 13.4216463

H 7.9618954 5.3061872 13.2165304

H 6.5514250 4.3549198 12.6974727

C 8.0770081 1.3987814 8.2497313

C 9.1094578 1.1235803 7.3692849

C 10.2477813 1.9121083 7.4098430

C 10.3066642 2.9467723 8.3265001

C 8.2215728 2.4538566 9.1347633

H 7.1708425 0.8065298 8.2590527

H 9.0300261 0.3045703 6.6637133

H 11.0831518 1.7305107 6.7456161

H 11.1781570 3.5850422 8.4147176

H 7.4565103 2.7098854 9.8585292

N 9.3122278 3.2141624 9.1696305

**1b·2b**

Zr 9.4563849 5.4376743 10.5757281

O 8.8786878 6.2650654 8.9049994

O 8.0680386 4.4327653 11.5543293

O 9.6280577 6.9435109 11.7792777

O 11.3336144 4.8236988 10.6325187

C 8.4878296 6.3660119 7.5847251

H 8.4916089 5.3856704 7.0864331

H 7.4734091 6.7767917 7.5039579

H 9.1611134 7.0268273 7.0242547

C 9.7175033 8.0975883 12.5327430

H 8.9090347 8.1481751 13.2727421

H 10.6705188 8.1423167 13.0745995

H 9.6483119 8.9891468 11.8973181

C 12.5351350 5.0367160 11.2941711

H 12.8425154 4.1397660 11.8472701

H 13.3350315 5.2783316 10.5823110

H 12.4650655 5.8654618 12.0112495

C 7.3896361 4.3489434 12.7619846

H 7.6003877 3.3960452 13.2646856

H 7.6734020 5.1592848 13.4464657

H 6.3048349 4.4090882 12.6051407

C 7.9698323 1.7426985 8.1078482

C 9.0470472 1.3464434 7.3327675

C 10.2565649 2.0032358 7.4866317

C 10.3412021 3.0331939 8.4069782

C 8.1429655 2.7827279 9.0039912

H 7.0069427 1.2547225 8.0266854

H 8.9461221 0.5348318 6.6214564

H 11.1274786 1.7241244 6.9074130

H 11.2648687 3.5721281 8.5846915

H 7.3417015 3.1250672 9.6488077

N 9.3043153 3.4182752 9.1487127

**1c·2b**

Hf 9.4680887 5.4065044 10.5840140

O 8.8936610 6.2574059 8.9223462

O 8.0849015 4.3914344 11.5649901

O 9.6218669 6.9142437 11.7896635

O 11.3463308 4.7937736 10.6460316

C 8.4999691 6.3789444 7.6056234

H 8.4727584 5.4031580 7.0986841

H 7.4974226 6.8194073 7.5319433

H 9.1885273 7.0244872 7.0456980

C 9.6660643 8.1100566 12.4777497

H 8.8522277 8.1738064 13.2111088

H 10.6134009 8.2181815 13.0207932

H 9.5698632 8.9638945 11.7952721

C 12.5445343 5.0045126 11.3141547

H 12.8464020 4.1074256 11.8701565

H 13.3496211 5.2422770 10.6068318

H 12.4738038 5.8342378 12.0299135

C 7.4150223 4.3456652 12.7799439

H 7.6597045 3.4265238 13.3280247

H 7.6735114 5.1977113 13.4224012

H 6.3280791 4.3608581 12.6272336

C 7.9677707 1.7576770 8.0997658

C 9.0428273 1.3636075 7.3206720

C 10.2556481 2.0126008 7.4809622

C 10.3461054 3.0328315 8.4111293

C 8.1457919 2.7872742 9.0065107

H 7.0022920 1.2757866 8.0139706

H 8.9376390 0.5597592 6.6012474

H 11.1252026 1.7347810 6.8992146

H 11.2723508 3.5652423 8.5939053

H 7.3469039 3.1264271 9.6556369

N 9.3108694 3.4158328 9.1572960

**1a·2c**

Ti 9.4295099 5.1205963 10.4719677

O 8.8189720 5.7710930 8.8655377

O 8.2505260 4.2035669 11.5334754

O 9.4567111 6.6655646 11.3684592

O 11.2103688 4.7044580 10.6009939

C 8.5462732 7.0444683 8.3805012

H 7.8384342 6.9840879 7.5448669

H 8.1030559 7.6890888 9.1501365

H 9.4566725 7.5352997 8.0127719

C 9.5427989 7.9717012 11.8147963

H 8.5553739 8.4479975 11.8200030

H 9.9407948 8.0057873 12.8356368

H 10.2035655 8.5642548 11.1709830

C 12.3063020 5.2454040 11.2617163

H 12.6580357 4.5680466 12.0502450

H 13.1352038 5.4045093 10.5609815

H 12.0649768 6.2092174 11.7275762

C 7.5436725 4.4954941 12.6940462

H 7.8163447 3.7985912 13.4962743

H 7.7468915 5.5152372 13.0447899

H 6.4643136 4.4034686 12.5200776

N 9.3907307 3.0915996 9.2425407

C 9.8457448 2.0055681 10.1057733

H 10.8585937 2.2203825 10.4451891

H 9.1882730 1.9385728 10.9717272

H 9.8379122 1.0479447 9.5665225

C 10.2767575 3.2109202 8.0883071

H 9.9380235 4.0356938 7.4625048

H 11.2878307 3.4217004 8.4352469

H 10.2793579 2.2827886 7.4999103

C 8.0253431 2.8307246 8.7956706

H 7.3735613 2.7560076 9.6656001

H 7.6899688 3.6584872 8.1718496

H 7.9738148 1.8958239 8.2204688

**1b·2c**

Zr 9.4435056 5.2736565 10.5009791

O 8.8257955 5.9308683 8.7484261

O 8.1383323 4.3187170 11.6250879

O 9.4880796 6.9583374 11.4461097

O 11.3448468 4.7851639 10.6804306

C 8.4441644 7.0382772 8.0063267

H 7.4293977 6.9119333 7.6071508

H 8.4542290 7.9551975 8.6104043

H 9.1192051 7.1916162 7.1543487

C 9.5373544 8.1648900 12.1197887

H 8.5334994 8.5903540 12.2384130

H 9.9705518 8.0396995 13.1196054

H 10.1502510 8.8941561 11.5761628

C 12.5894372 5.1474075 11.1724703

H 12.9632088 4.3989501 11.8832968

H 13.3237077 5.2301474 10.3604342

H 12.5553827 6.1141832 11.6921002

C 7.3173857 4.3382252 12.7417340

H 7.6083817 3.5579052 13.4568898

H 7.3644256 5.3037054 13.2625054

H 6.2709322 4.1595513 12.4622865

N 9.4057447 3.0983890 9.2642869

C 9.8306569 2.0340961 10.1699687

H 10.8367809 2.2502298 10.5295383

H 9.1515132 1.9968600 11.0217509

H 9.8284480 1.0597989 9.6611884

C 10.3200648 3.1835240 8.1285256

H 10.0010267 3.9933581 7.4725280

H 11.3243098 3.3990920 8.4934716

H 10.3338150 2.2413152 7.5627944

C 8.0471882 2.8409084 8.7942172

H 7.3729317 2.8041187 9.6501521

H 7.7371568 3.6513851 8.1347539

H 7.9929985 1.8881293 8.2490206

**1c·2c**

Hf 9.4442548 5.2533285 10.4866928

O 8.8281103 5.9176698 8.7334588

O 8.1388414 4.2953441 11.6139080

O 9.4841255 6.9347760 11.4397512

O 11.3479624 4.7649029 10.6678494

C 8.4450273 7.0421698 8.0178803

H 7.4309286 6.9234417 7.6145492

H 8.4526082 7.9454008 8.6423096

H 9.1199585 7.2169252 7.1699065

C 9.5290928 8.1368846 12.1195415

H 8.5242297 8.5598085 12.2403075

H 9.9618282 8.0090985 13.1193808

H 10.1399601 8.8719376 11.5812027

C 12.5787996 5.1547376 11.1737135

H 12.9640249 4.4119698 11.8844669

H 13.3188446 5.2580482 10.3694156

H 12.5186807 6.1180290 11.6973741

C 7.3328568 4.3682792 12.7398754

H 7.6159750 3.6041153 13.4753414

H 7.4060280 5.3478401 13.2305206

H 6.2793404 4.2029168 12.4793917

N 9.4061411 3.1004226 9.2647631

C 9.8289984 2.0315122 10.1689807

H 10.8340213 2.2470546 10.5314925

H 9.1471430 1.9908973 11.0181492

H 9.8280424 1.0607149 9.6544715

C 10.3228267 3.1803860 8.1282605

H 10.0072259 3.9905250 7.4713292

H 11.3275515 3.3912121 8.4939896

H 10.3306905 2.2367760 7.5656397

C 8.0482887 2.8397075 8.7889500

H 7.3714358 2.7999243 9.6424629

H 7.7393951 3.6498999 8.1288394

H 8.0014709 1.8874902 8.2429811

**S7. References**

[1] **TURBOMOLE V7.7**, TURBOMOLE GmbH, Karlsruhe, 2021.

[2] Ahlrichs, R.; Bär, M.; Häser, M.; Horn, H.; Kölmel, C. *Chem. Phys. Lett.* **1989**, *162*, 165–169.

[3] Furche, F.; Krull, B. T.; Nguyen, B. D.; Kwon, *WIREs Comput. Mol. Sci.* **2014**, *4*, 91–100.

[4] Adamo, C.; Barone, V. *J. Chem. Phys.* **1999**, *110*, 6158–6170.

[5] Caldeweyher, E.; Bannwarth, C.; Grimme, S. *J. Chem. Phys.* **2019**, *150*, 154122.

[6] Weigend, F.; Ahlrichs, R. *Phys. Chem. Chem. Phys.* **2005**, *7*, 3297–3305.

[7] Kitaura, K.; Morokuma, K. *Int. J. Quantum Chem.* **1976**, *10*, 325.
[8] Ziegler, T.; Rauk, A. *Theor. Chim. Acta* **1977**, *46*, 1.

[9] Lu, T.; Chen, F. *J. Comput. Chem.* **2012**, *33*, 580–592.

[10] Humphrey, W.; Dalke, A.; Schulten, K. *J. Mol. Graph.* **1996**, *14*, 33–38.

[11] Glendening, E. D.; Landis, C. R.; Weinhold, F.*NBO 7.0*, Theoretical Chemistry Institute, University of Wisconsin, Madison, 2018.

[12] Dennington, R.; Keith, T.; Millam, J. *GaussView, Version 6.1.1*; Semichem Inc.: Shawnee Mission, KS, 2016.

[13] Sheldrick, G. M. *Acta Crystallogr. Sect. A Found. Adv.* **2015**, *71*, 3–8.

[14] Sheldrick, G. M. *Acta Crystallogr. Sect. C Struct. Chem.* **2015**, 71, 3–8.

[15] Spek, A. L. *Acta Crystallogr. Sect. D Biol. Crystallogr.* **2009**, *65*, 148–155.
